# Knowledge, Concerns, and Psychological Distress Among Caregivers of Patients Seeking Emergency Medical Care for Non-COVID-19-Related Illness During the COVID-19 Pandemic: A Cross-Sectional Study

**DOI:** 10.7759/cureus.45115

**Published:** 2023-09-12

**Authors:** Niraj Srivastava, Varun Anand, Ajay Kumar, Sunita Singh, Chandan Dey, Santosh K Rathia

**Affiliations:** 1 General Surgery, All India Institute of Medical Sciences, Raebareli, Raebareli, IND; 2 Trauma and Emergency/Pediatric Emergency Medicine, All India Institute of Medical Sciences, Raipur, Raipur, IND; 3 Psychiatry, All India Institute of Medical Sciences, Raipur, Raipur, IND; 4 General Surgery/Pediatric Surgery, All India Institute of Medical Sciences, Raebareli, Raebareli, IND; 5 Emergency Medicine and Trauma, All India Institute of Medical Sciences, Raipur, Raipur, IND

**Keywords:** non-covid-19 illness, emergency medical services, covid-19 peritraumatic distress index, cpdi, stress or psychological distress, awareness and concerns, coronavirus, covid-19 pandemic

## Abstract

Introduction

During the active phase of the COVID-19 pandemic, the global healthcare system failed to meet the increased demand for healthcare resources, infrastructures, and facilities. The brunt of the healthcare crisis was faced not only by COVID-19 victims; a large majority of non-COVID patients were deprived of routine and emergency care. Factors that possibly affected resource utilization, healthcare-seeking behavior, service delivery patterns, and national health systems’ priority during the pandemic were the knowledge and attitudinal concerns related to the COVID-19 disease and its control measures. Here, we evaluated the knowledge, concern, and psychological distress among the caregivers of the patients attending the emergency department at a tertiary healthcare center in India.​

Methodology

We conducted a survey-based study using a pre-validated questionnaire on the caregivers of the patients visiting the emergency department (ED) from June to September 2020 (during the first wave of the COVID-19 pandemic). The demographic details and responses of the participants were documented in the semi-structured proforma. A pre-validated COVID-19 Peritraumatic Distress Index (CPDI) questionnaire was used to assess psychological stress.

Results

Out of 1014 participants interviewed, the majority were male attendants (72%), aged 18-45 (82%), and seeking medical attention for patients with chronic illnesses (76%). Acute onset emergencies like stroke, myocardial infarction, trauma, etc. were the ED presentation in only one-fifth of patients. COVID-19-related knowledge was adequate for questions related to age groups at risk for the viral infection (97% agreed that all age groups were at risk), mode of transmission (75-90% were aware of the common modes of transmission), and >65% knew the common symptoms of COVID-19 infection. However, only 38.5% knew about frequent handwashing as a protective measure. More than half of the participants considered the COVID-19 vaccine as the sole ray of hope and disregarded the effectiveness of alternative medicines such as Ayurvedic/homeopathic/allopathic medicines as preventive options. One-third were first-time visitors to the hospital, while two-thirds of all participants were afraid to visit any hospital during the COVID-19 pandemic. The majority (84%) faced difficulty in accessing the index tertiary care center due to transport, socioeconomic support, or lockdown-related restrictions. In comparison, 60% reported some form of discrimination at almost all levels of healthcare settings due to COVID-19-related priority changes. Nearly half (48%) of all enrolled caregivers reported experiencing mild-to-moderate distress (CPDI score=28-51), and 15.7% felt severe distress (CPDI score >51) while seeking treatment for the non-COVID-19 illness of their patient. Age and socioeconomic status were significantly associated with COVID-19-related psychological distress levels (p<0.001 in logistic regression), while gender, education, and residence showed no significant associations.

Conclusion

Most of the patient caregivers visiting the emergency department during the COVID-19 pandemic had an adequate understanding of risk factors and preventive measures. The major barriers to accessing healthcare facilities were transport, financial issues, and lockdown-related restrictions. Almost two-thirds of the caregivers revealed mild-to-moderate to severe psychological distress due to the pandemic and lockdown-related concerns.

## Introduction

Amidst the chaos and crisis situation of the recent COVID-19 pandemic in 2020-2021, most government hospitals in India became busy with the management of COVID-19 patients. Non-COVID-19 but acute emergency patients and those with chronic illnesses that require regular hospital visits faced multiple hurdles in accessing the required medical care. Emergency and trauma patients, pregnant women, extreme age patients (e.g., elderly and children), those with chronic comorbidities (e.g., chronic kidney, liver, lung, or heart disease), and cancer patients requiring ongoing therapies found it daunting to reach hospitals. This healthcare crisis affected both COVID-19 and non-COVID-19 patient management. Such reports have been prevalent in the news and social media, not only in India but in almost all countries since the global outbreak was declared. The healthcare service system and authorities had a tremendous challenge in responding with prompt, effective, and appropriate interventions, policies, and public messages. The pandemic and its restrictions, including the lockdown, might have significantly affected mental and physical well-being, social cohesion, economic stability, and trust in oneself, others, and the government’s medical system.

This survey targeted the relatives or attendants taking care of patients at an ED during the COVID-19 pandemic and assessed their knowledge, concerns, and psychological distress related to the pandemic, healthcare system crisis, and resource limitations. The in-depth interview proforma included a questionnaire related to almost all possible hindrances in seeking emergency treatment for acute or chronic non-COVID-19 illnesses. The study aimed to involve at least 1,000 participants to attain an adequate sample size for such a behavior-based survey [1]. After exploring the basic knowledge/awareness, and problem concerns in terms of personal, socioeconomic, physical, and health-infrastructural inconveniences, the level of psychological or mental stress was evaluated using the CPDI (COVID-19 Peritraumatic Distress Index) score [2].

## Materials and methods

Study design: Cross-sectional interview-based study

Study setting: Department of Trauma and Emergency, AIIMS, Raipur, India

Study duration: Four months after IEC clearance

Participants

Inclusion criteria: Any caregiver of a patient with an age >18 years

Exclusion criteria: Incompletely filled questionnaire, negative consent for participation

Sample size: A sample size adequately representative of age, gender, and locality was a minimum number of 1,000 participants for such an analytical survey as suggested by a "survey tool and guidance" released during the COVID-19 pandemic by WHO’s regional office for Europe in July 2020 [1]. We also used a sample size of 1,000 to make the study sample as representative as possible of the study population, and the target data could be collected over 4 months during the first wave of the COVID-19 pandemic in India (June to September 2020).

Brief methodology and data collection

After clearance of the study proposal from the institutional research cell and ethics committee, the predesigned case proforma and survey questionnaire were finalized by all investigators and validated by two independent experts familiar with the terminology and basics of COVID-19, behavioral science, and interview skills. The Delphi method with e-mail communications was used to take and share inputs and suggestions on all the parts and elements of the questionnaire for needed modifications. Two independent language experts reviewed the Hindi version of the questionnaire to validate the correct English-to-Hindi translation and vice versa.

The study questionnaire framed was in three parts:

Part 1: Sociodemographic details and basic COVID-19 knowledge/awareness questionnaire.

Part 2: Eleven questions related to physical, personal, social, and economic inconvenience for seeking medical care and attitude towards services.

Part 3: The questionnaire incorporated a context-specific tool for diagnosing acute stress disorders, also known as the CPDI [2], which inquired about the frequency of anxiety, depression, specific phobias, cognitive change, avoidance, and compulsive behavior, physical symptoms and loss of social functioning in the past week, with a total score ranging from 0 to 96. A score between 28 and 51 indicates mild-to-moderate distress, and a score >52 indicates severe distress. Psychiatrists from the Shanghai Mental Health Centre had already verified the content validity of the CPDI. The Cronbach’s alpha of the CPDI questionnaire was ≥0.92 in the Chinese study, which had initially developed the CPDI as a COVID-19-specific scoring tool [2], as well as in an Indian validation study [3]. The Cronbach alpha value of >0.90 is taken as a good measure of reliability or internal consistency used for the validation of any questionnaire. In our study setting, on initial piloting of the first 30 participants, Cronbach’s alpha obtained for the CPDI questionnaire was 0.922, which assured its reliability and applicability for further data collection.

During the study period, at least one of the relatives of each admitted non-COVID-19 patient was screened for eligibility, and the consenting participants were interviewed in the emergency department mostly in the waiting area. The interviewers maintained appropriate social and physical distance from the patients and participants as per the COVID-19-appropriate behavior norms and institutional guidelines. The modified Prasad classification (MPC) was used to estimate the socioeconomic status of the participants' families. Incomplete proforma were excluded from data compilation. The whole data were entered and compiled in an Microsoft Excel spreadsheet to be transformed into statistical form.

Outcome variables and statistical analysis: Data analysis was performed using SPSS statistical software (version 20.0). There was descriptive data generation for the demographic profile of participants. Continuous or numerical data were represented in mean and SD, while the chi-square test was applied to test the difference in categorical variables like knowledge or awareness levels and concerns or problems in seeking emergency medical treatment. Their perception toward the government or healthcare system by age, gender, education status, rural or urban locality, and socioeconomic status were presented in frequency, percentage, or proportion. Psychological distress during treatment for non-COVID-19 illnesses was assessed by the CPDI [2]. A p-value of less than 0.05 was considered statistically significant for all analytical interpretations.

Ethical issues: The study was initiated only after approval from the Institute Ethics Committee (Letter no. 1121/IEC-AIIMSRPR/2020, dated: 22/06/2020). Data from the participants was collected anonymously. Data was obtained only from adult respondents or participants aged >18 years after they signed informed consent.

## Results

The study included 1014 participants, with 72% being male relatives attending the patients in the ED and only 28% female attendants. At the index referral hospital, two-thirds of patients and participants came from urban areas, while 37.3% were from distant rural areas.

Table 1 shows the demographic distribution of the 1,014 participants*:* The majority (86%) were adults aged between 18 to 45 years, and 94% were educated up to schooling or graduation. However, 2.1% (21 attendants) were elderly with ages above 60 years, and 6% were illiterate too. Occupation-wise, half of the participants were self-employed or worked in agriculture, and 12% were unemployed adults. Family structure-wise, the majority (62%) belonged to nuclear families. With respect to monthly family income, 34% earned below INR 13,160, and only 4% had income above INR 52,734. As per the modified Prasad classification (MPC), the majority (>75%) belonged to the middle class (II, III). Nearly 90% of patient attendants were family members or close relatives, while only 10-11% were neighbors, friends, or others.

**Table 1:** Demographic profile of the study participants

Demographic Variables	Value or Category	Number	Percent (%)
Age group (in years)	18-30	367	36.2
	31-45	506	49.9
	46-60	120	11.8
	>60	21	2.1
Gender	Male	729	71.9
	Female	285	28.1
Education	Illiterate	63	6.2
	Primary school	30	3.0
	High school	525	51.8
	Graduate	304	30.0
	Postgraduate	92	9.1
Occupation	Government job	78	7.7
	Nongovernment job	216	21.3
	Own business/farming	499	49.2
	Student	98	9.7
	Unemployed	123	12.1
Family income (in rupees per month)	2,640	37	3.6
	2,641-7,886	72	7.1
	7,887-13,160	238	23.5
	13,161-19,758	202	19.9
	19,759-26,354	194	19.1
	26,355-52,733	231	22.8
	≥52,734	40	3.9
SES (socioeconomic status)	I (Upper)	77	7.6
	II (Upper middle)	483	47.6
	III (Lower middle)	292	28.8
	IV (Upper lower)	79	7.8
	V (Lower)	83	8.2
Total number of family members	<4	422	41.6
	4-8	538	53.1
	>8	54	5.3
Family type	Joint family	381	37.6
	Nuclear family	633	62.4
Residence	Rural	378	37.3
	Urban	636	62.7
Type of disease	Sudden-onset illness (injury/heart attack/paralysis, etc.)	242	23.9
	Chronic disease (follow-up)	584	57.6
	Acute or chronic disease flare-up (in which the patient is unable to perform routine tasks)	188	18.5
Relationship with the patient	Son/Daughter/Daughter-in-law/Son-in-law/Nephew/Niece	236	23.3
	Brother/Sister/Spouse	280	27.6
	Father/Mother/Grandfather/Grandmother	388	38.3
	Neighbors/Friends	83	8.2
	Others	27	2.7

During the study period, which coincided with the peak of the first wave of the COVID-19 pandemic, the majority of patients (76%) visiting the ED had chronic uncontrolled diseases or experiencing acute flare-ups. Less than one-fourth of patients presented with acute illnesses such as myocardial infarction, stroke, sudden paralysis, or traumatic injuries.

Basic knowledge and awareness regarding the coronavirus disease and COVID-19 pandemic

In this study, the participants were assessed for basic awareness about the disease, its spread, and preventive measures. The majority (97%) of them were aware that people of all age groups were at risk of coronavirus infection. More than 75% of patient attendants knew about the common modes of coronavirus spread (e.g., via air droplets (89%), touching infected things or people (75%), contact with infected people coughing, sneezing, or breathing (83%)).

Regarding the main symptoms or signs of COVID-19 infection, four major clinical features were agreed upon by most responders (i.e., fever (93.4%), shortness of breath (88.5%), sore throat and/or nose block (80%), and excessive fatigue or muscle pain (65%)). However, about reduced smelling sense, around 70% responded either "no" or "don’t know," and in the same way, >60% were unaware of gastrointestinal manifestations of this viral disease like diarrhea.

With regard to preventive measures (Table 2), the majority knew about and agreed on three common measures, which were already proven and advocated prophylactic interventions like not touching the face and mouth/nose repeatedly (74%), covering the mouth and nose with a cloth or mask (66%), and maintaining a distance of 2 yards (66%). However, one of the most standard steps in personal protective measures (i.e., “frequent handwashing with soap") was ticked “yes” by only 38.5%, and 10% of participants ticked "don’t know.” Until then, the COVID-19 vaccine was not that established for efficacy and safety, but surprisingly, 56% of participants marked it as a new hope at that time. For Ayurvedic/homeopathic and allopathic medicines, only 37% and 35% considered them as preventive options, respectively, and the remaining majority voted against them. Two-thirds (67%) of participants either agreed on the non-availability of other new and effective preventive or therapeutic options or said they didn’t know about more alternatives available at that time.

**Table 2:** Participants’ opinions about preventive measures against coronavirus infection

In your opinion, which of the following measures could be taken to prevent coronavirus infection?						
Measures	Yes		No		Don’t know	
	N	%	N	%	N	%
Frequently washing hands with soap	390	38.5	527	52.0	97	9.6
Not touching the face	751	74.1	239	23.6	24	2.4
Covering the mouth and nose with a piece of cloth	669	66.0	313	30.9	32	3.2
Keeping a distance of two yards	672	66.3	284	28.0	58	5.7
Vaccination for prevention	567	55.9	383	37.8	64	6.3
Using Ayurvedic/homeopathic medicine	380	37.5	551	54.3	83	8.3
Using allopathy medicine	349	34.4	575	56.7	90	8.9
Apart from this, is there any other way of treatment in your understanding?	330	32.5	634	62.5	50	4.9

Assessment of problems related to seeking treatment from the hospital ED during the COVID-19 pandemic

Of all 1014 participants, 71% had experienced getting some treatment from AIIMS Raipur Hospital earlier, and the remaining 29% were visiting this hospital or ED for the first time. Only 5% of caregiver participants were aware of the availability of separate dedicated COVID-19 screening cum treatment areas. Notably, two-thirds (66%) of attendants reported that they were afraid to come to this tertiary hospital during the COVID-19 pandemic. The concern and level of difficulty in reaching or accessing this hospital was high as reported by the study participants; 56% considered it difficult, and 27.3% mentioned it as very difficult. The main reasons given for the difficulty in reaching this hospital were the nonavailability of public or private transport because of COVID-19 pandemic constraints (52%), lack of money (25%), and poor socio-familial support (21.4%). Few (18.6%) also showed concerns about difficult access to desired services during that crisis time.

With regard to problems faced during the treatment of their current patient, the following were the major perceptions and concerns of the patient's attendants/relatives:

1. The severity category of the brought patient was mostly serious (52%) or very serious (5%), compared to less urgent or chronic conditions (43%).

2. Among the patients brought here, 43% had already visited or consulted with some other physician or hospital before coming to AIIMS hospital.

3. In 55% of cases, the reason for bringing the patient to AIIMS hospital was a referral by an outside doctor or hospital. Conversely, a lack of funds and the unavailability of necessary resources at the prior hospital as individual factors compelled the family in >25% of cases.

4. During the COVID-19 pandemic, nearly one-fourth of participants reported that other nearby healthcare services (especially private clinics and hospitals) were not as functional or available as before in their locality.

5. The majority (66.7%) of patients had been brought from a COVID-19-infected area.

6. Sixty percent of patients or their relatives revealed that they experienced some direct or indirect discrimination by the staff or doctors while referring or first attending at a health setup due to COVID-19-related priority shifts and nondeliberate neglect of routine healthcare services.

7. More than half (58.4%) of participants accepted that there was a delay in bringing their patient to AIIMS hospital, and the main reasons for the delay were misinterpreting the patient's condition as stable and staying at home for a few more days (in 34.3% cases), lack of proper transport and conveyance facilities (20%), and being afraid of acquiring coronavirus infection at such high-load centers for COVID-19 care in central India (20%).

8. The majority (80.5%) of participants agreed that they were afraid of going out of their house (even to seek medical services) during lockdown restrictions.

9. Almost two-thirds of them (63.6%) faced difficulties due to maintaining social distancing during various steps of patient treatment and admission in the hospital (e.g., long queues for registering at the emergency department, billing, investigations, purchasing medicines, etc.).

10. In terms of satisfaction of the participants, 66% showed complete satisfaction, 28% had partial satisfaction, and only 6% were not satisfied with the health services during the pandemic crisis.

Assessment of psychological stress amongst the patient-attendants using the COVID-19 Peritraumatic Distress Index (CPDI) questionnaire

Table 3 shows the profile of actual responses to each of the 24 questions asked to assess the stress level among the study participants using the CPDI scoring system.

**Table 3:** Evaluation of psychological distress using the CPDI questionnaire and score Q. No.: Question Number; CPDI: COVID-19 Peritraumatic Distress Index

Q. No.	CPDI-specific question	Participants' Response (with CPDI Score)				
		Never - 0	Occasionally - 1	Sometimes - 2	Often - 3	Most Often - 4
1	Compared to usual, I feel more nervous and anxious.	505 (49.8%)	214 (21.1%)	179 (17.6%)	71 (7.0%)	46 (4.5%)
2.	I felt insecure and bought a lot of masks, medications, sanitizer, gloves, and/or other home supplies.	215 (21.2%)	451 (44.4%)	211 (20.8%)	85 (8.4%)	53 (5.2%)
3.	I can’t stop myself from imagining myself or my family being infected and feel terrified and anxious about it.	230 (22.7%)	201 (19.8%)	430 (42.4%)	105 (10.3%)	49 (4.8%)
4.	I feel empty and helpless no matter what I do.	249 (24.5%)	224 (22.1%)	246 (24.2%)	220 (21.7%)	76 (7.5%)
5.	I feel sympathetic to the COVID-19 patients and their families. I feel sad for them.	174 (17.1%)	170 (16.7%)	267 (26.3%)	195 (19.2%)	209 (20.6%)
6.	I feel helpless and angry about people around me, governors, and media.	235 (23.2%)	190 (18.7%)	273 (26.9%)	221 (21.8%)	96 (9.5%)
7.	I am losing faith in the people around me.	315 (31.0%)	210 (20.7%)	294 (29.0%)	116 (11.4%)	80 (7.9%)
8.	I collect information about COVID-19 all day. Even if it’s not necessary, I can’t stop myself.	453 (44.6%)	264 (26.0%)	175 (17.2%)	60 (5.9%)	63 (6.2%)
9.	I will believe the COVID-19 information from all sources without any evaluation.	330 (32.5%)	356 (35.1%)	232 (22.9%)	68 (6.7%)	29 (2.9%)
10.	I would rather believe in negative news about COVID-19 and be skeptical about the good news.	314 (30.9%)	209 (20.6%)	368 (36.3%)	83 (8.2%)	41 (4.0%)
11.	I am constantly sharing news about COVID-19 (mostly negative news).	319 (31.4%)	238 (23.4%)	232 (22.9%)	164 (16.2%)	62 (6.1%)
12.	I avoid watching COVID-19 news as I am too scared to do so.	312 (30.7%)	207 (20.4%)	271(26.7%)	126 (12.4%)	99 (9.8%)
13.	I am more irritable and have frequent conflicts with my family.	318 (31.3%)	210 (20.7%)	234 (23.1%)	175 (17.2%)	77 (7.6%)
14.	I feel tired and sometimes even exhausted.	292 (28.8%)	192 (18.9%)	282 (27.8%)	158 (15.6%)	90 (8.9%)
15.	Due to feelings of anxiety, my reactions are becoming sluggish.	310 (30.5%)	195 (19.2%)	270 (26.6%)	151 (14.9%)	89 (8.8%)
16.	I find it hard to concentrate.	317 (31.2%)	200 (19.7%)	260 (25.6%)	159 (15.7%)	79 (7.8%)
17.	I find it hard to make any decisions.	315 (31.0%)	213 (21.0%)	258 (25.4%)	151 (14.9%)	78 (7.7%)
18.	During this COVID-19 period, I often feel dizzy or have back pain and chest distress.	348 (34.3%)	195 (19.2%)	259 (25.5%)	136 (13.4%)	77 (7.6%)
19.	During this COVID-19 period, I often feel stomach pain, bloating, and other stomach discomfort.	422 (41.6%)	260 (25.6%)	214 (21.1%)	62 (6.1%)	57 (5.6%)
20.	I feel uncomfortable when communicating with others.	336 (33.1%)	253 (24.9%)	305 (30.0%)	85 (8.4%)	36 (3.5%)
21.	Recently, I have rarely talked to my family.	349 (34.4%)	179 (17.6%)	329 (32.4%)	123 (12.1%)	35 (3.4%)
22	I cannot sleep well. I always dream about myself or my family being infected by COVID-19.	315 (34.6%)	206 (20.3%)	283 (27.9%)	119 (11.7%)	56 (5.5%)
23.	I lost my appetite.	344 (33.9%)	236 (23.3%)	243 (23.9%)	130 (12.8%)	62 (6.1%)
24.	I have constipation or frequent urination.	362 (35.7%)	241 (23.7%)	238 (23.4%)	109 (10.7%)	65 (6.4%)

The CPDI scores (mean and standard deviation, SD) for each of the 24 questions have been represented in Table 4, which reveals that the varied responses of participants provided widely dispersed scores (out of 0-4) to almost all questions which were designed to address different aspects of mental and psychological stress.

**Table 4:** The mean score and SD values of responses against 24 questions of CPDI score Q. No.: Question number; CPDI: COVID-19 Peritraumatic Distress Index; SD: standard deviation

Q. No. of CPDI questionnaire	Mean score of study participants (N=1014)	SD
Q1	0.95	1.166
Q2	1.32	1.05
Q3	1.54	1.09
Q4	1.65	1.26
Q5	2.09	1.36
Q6	1.75	1.28
Q7	1.44	1.25
Q8	1.03	1.18
Q9	1.12	1.03
Q10	1.33	1.11
Q11	1.42	1.25
Q12	1.50	1.30
Q13	1.49	1.30
Q14	1.57	1.29
Q15	1.52	1.29
Q16	1.49	1.28
Q17	1.47	1.27
Q18	1.40	1.28
Q19	1.08	1.17
Q20	1.24	1.10
Q21	1.32	1.16
Q22	1.33	1.21
Q23	1.33	1.23
Q24	1.28	1.23

On categorizing the study participants based on the three levels of distress (Table 5), 36.3% reported feeling no to least distress, around half (48%) of them perceived mild-to-moderate levels of distress, and only 15.7% experienced severe distress during the COVID-19 pandemic while seeking their patient’s care.

**Table 5:** Distribution of psychological distress level among study participants CPDI: COVID-19 Peritraumatic Distress Index

Level of stress/distress {range of CPDI score}	Number of participants (N=1014)	Percentage
No distress (0-28)	368	36.3%
Mild-to-moderate distress (29-51)	487	48.0%
Severe distress (>51)	159	15.7%

Table 6 shows that no significant association of level of stress was seen with gender and rural or urban residence, whereas a significant association was seen (p<0.001) with age group, education, and SES.

**Table 6:** Association of demographic characteristics with different levels of stress or psychological distress SES: Socioeconomic Status **: Significant

Demographic Variables		No Distress (N=368)		Mild-to-Moderate Distress (N=487)		Severe Distress (N=159)		P value
		n	%	n	%	n	%	
Gender	Male	252	68.5%	358	73.5%	119	74.8%	0.179
	Female	116	31.5%	129	26.5%	40	25.2%	
Age group	18-30 years	156	42.4%	141	29.0%	70	44.0%	<0.001**
	31-45 years	149	40.5%	274	56.3%	82	52.2%	
	46-60 years	52	14.1%	62	12.7%	6	3.8%	
	>60 years	11	3.0%	10	2.1%	0	0.0%	
Education	Illiterate	32	8.7%	25	5.13%	6	3.77%	<0.001**
	Primary school	21	5.7%	5	1.02%	4	2.51%	
	High school	164	44.6%	276	56.67%	85	53.45%	
	Graduate	121	32.9%	141	28.95%	42	26.41%	
	Postgraduate	30	8.2%	39	8%	23	14.46%	
Residence	Rural	137	37.22%	172	35.31%	69	43.39%	0.270
	Urban	230	62.5%	316	64.88%	90	56.60%	
SES	I (Upper)	45	12.22%	18	3.69%	14	8.80%	<0.001**
	II (Upper middle)	132	35.86%	261	53.59%	90	56.60%	
	III (Lower middle)	107	29.07%	148	30.39%	36	22.64%	
	IV (Upper lower)	43	11.68%	27	5.54%	10	6.28%	
	V (Lower)	41	11.14%	33	6.77%	9	5.66%	

From binomial logistic regression analysis (Table 7), it was found that, compared to no stress, the odds of having mild-moderate to severe stress are significantly higher among the younger age group <45 years (OR=1.515, p=0.024). In relation to SES, the upper class had higher odds (OR=1.613, p<0.001) of having moderate-to-severe stress than the lower class, which was highly significant. The same comparative results were nonsignificant for gender, education, and residence.

**Table 7:** Binomial logistic regression to assess demographic factors affecting stress level (no or least stress versus mild-to-moderate and severe stress)

Variables		Odds Ratio	95% CI	P value
Gender	Males	1.308	0.58-1.025	0.062
	Females			
Age group	<45 years	1.515	0.693-0.999	0.024
	>45 years			
Education	Till School	0.937	0.856-1.026	0.229
	Graduate or more			
Residence	Rural	0.993	0.865-1.140	0.860
	Urban			
Socioeconomic status/class	Upper class (upper and upper-middle)	1.613	0.703-0.916	<0.001
	Lower class (lower-middle and lower classes)			

## Discussion

WHO declared the COVID-19 outbreak as a global health emergency on January 31, 2020, due to high viral transmission rates, severity, and mortality [2]. This recent pandemic recalled the dreadful impact of the prior SARS outbreak of 2002 [4]. The case fatality rate of COVID-19 varied across different age groups and countries. It was initially reported at 6.3%, but it reached over 15% in certain locations based on varying caseloads and mortality reporting [5]. The pandemic also increased stress and anxiety among the public, and as the new control measures such as quarantine and home or facility-based isolation were introduced, loneliness, depression, alcohol and substance abuse, and self-harm or suicidal behavior were likely to rise as well [6].

The ED of the index hospital was directly involved in managing both non-COVID-19 and suspected COVID-19 emergencies, which posed significant stress along with the risk of contracting COVID-19 infection among the attendants of the patients. This study aimed to assess the basic awareness, concerns, and variables that could characterize and determine the level of psychological distress amongst the attendants of patients suffering from illnesses other than COVID-19 during the pandemic.

Among the cohort of 1,014 participants, a significant proportion, close to 90%, were found to be the familial or intimate associates of the patients, while the remaining were neighbors, friends, or other acquaintances. More than two-thirds were male, and a similar proportion of them hailed from urban or suburban residences and belonged to middle socioeconomic status. The majority (around 90%) were young adults aged 18-45 years and were educated at least up to primary schooling.

During a pandemic, the risk communication to ensure timely and effective public health awareness can be achieved not only by disseminating knowledge through literature such as books, articles, internet sources, and prevalent guidelines or tabletop emergency preparedness policies but also by proactive capacity building and encouraging the healthcare providers to take the new role as first emergency responders to the victims [7]. The term "healthy psychology" encompasses psychological and psychosocial factors such as life satisfaction, optimism, self-esteem, and social support perception. Conversely, undesirable psychological states such as anxiety, stress, depression, and hostility may impact the overall disease course, as well as the patient’s recovery and survival [8]. These same factors might apply to caregivers or relatives during such a mass crisis as the COVID-19 pandemic. Understanding and supporting the psychological well-being of both patients and caregivers are crucial aspects of comprehensive healthcare during such critical situations. Contrary to the common belief that people typically panic during acute crises, the initial response to the first wave of the COVID-19 pandemic was variable worldwide, though it ultimately provoked widespread panic. Additionally, individuals may display more compassionate and voluntary behavior during and after a disaster [7]. However, in severe crises, a significant proportion of those exposed may experience mental stress disorders, manifested through depression, loneliness, difficulty coping, anger, emotional instability, physical debilitation, psychological distress, and impaired decision-making and information processing. While these symptoms are often transient, in some cases they may persist long-term, leading to post-traumatic stress disorder (PTSD) after the immediate crisis subsides.

During the COVID-19 outbreak, individuals still fell ill from non-COVID-19-related diseases, necessitating emergency care that did not receive the same level of attention as the global pandemic's priority spotlight. Like many other countries, hundreds of indicators showed a worrying disruption in India’s essential health services during the pandemic as the focus of both central and local administrations was narrowed to containment of the coronavirus. The consequences were evident in several health service indicators, including curtailed routine immunization schedules, limited access to inpatient, outpatient, and ED treatment of both infectious and noncommunicable diseases, reduced laboratory investigations, and restricted availability of mental healthcare [9]. An analytical report on health burden released in March 2020 by the National Health Mission (NHM), Govt. of India (GoI) represented 150,000 health facilities across 627 districts, which meant that data for roughly 40,000 facilities from the remaining 75 districts could not be reported presumably due to lockdown-related disruptions in the administrative machinery. Services for pregnant women, such as iron and calcium supplements and tetanus injections, were largely maintained, but medical interventions for pregnant women saw a sharp decline, potentially leading to more unattended home births. Additionally, a large proportion of children missed essential and routine vaccinations. A significant reduction was notified with respect to outpatient treatment visits in 2020 for diabetes, mental illness, and cancer, with nearly 350,000, 150,000, and 100,000 fewer visits, respectively, compared to the same March month of 2019. Similarly, a month-to-month reduction in acute cardiac, obstetric, and cerebrovascular emergencies of 1.2%, 2.1%, and 5.3%, respectively, presumably suggested limited healthcare access due to lockdown and transport restrictions. In the current study, too, most patients visiting the ED had either chronic diseases and their flare-ups (76%) or acute time-sensitive emergencies (24%). We excluded the confirmed cases of acute COVID-19 pneumonia cases and also the suspected cases, which could be directly sent or shifted to the separate and dedicated COVID-19 screening-cum-treatment area. Additionally, the selective entry of patients with chronic multi-morbid conditions into this tertiary-level government hospital during the health crisis could be attributed to the limited acceptance of such patients in private and peripheral government hospitals.

During the Severe Acute Respiratory Syndrome (SARS) pandemic in 2002-2003, studies showed that people were more compliant with health recommendations once they were convinced about the disease severity. Moreover, they believed the recommendations were effective and that the government provided transparent and trustworthy information about the outbreak fostering confidence in controlling the spread of the infection [10]. Moreover, higher levels of anxiety or worry during SARS were associated with increased behavior changes and pre-existing emotional states which affect an individual’s ability to process new information and cope with unusual circumstances [11]. Also, during the initial 3 months of the recent COVID-19 pandemic, distressing information about daily deaths, isolation, fear of the novel or mutant virus, and even social media rumors or misinformation generated widespread psychological trauma. US Federal agencies and experts warned about a historic wave of mental health problems, including depression, substance abuse, PTSD, and suicide [12]. Compared to previous pandemics, COVID-19 appeared to cause higher suicidality [13, 14]. In India, the national lockdown imposed on March 24, 2020, resulted in a surge in mental illness cases and suicidal tendencies. Similar alarming reports of psychological distress were also observed in countries like Bangladesh [15]. Reasons for COVID-19-related suicides included fear of the disease, fear of transmission, mental instability due to quarantine or isolation, economic hardships, and lack of social-familial support [13,16,17]. In the Chinese study determining the impact of stringent quarantine on the prevalence of anxiety and depression, it was significantly higher in the affected group (12.9%, 22.4%) than in the unaffected group (6.7%, 11.9%), respectively [17]. Lower family income, lower education level, higher self-evaluated level of knowledge, more worry about getting infected, having poor psychological support, greater property damage, and lower self-perceived health condition were significantly associated with higher scores on the self-reporting scores of anxiety (SAS) and depression (SDS). The Indian Psychiatry Society reported a 20% rise in mental illness cases with stringent lockdown measures as a significant contributing factor. Some individuals resorted to suicide before obtaining their COVID-19 test results in India [18,19]. In the current survey, a large majority (80.5%) of participants agreed that they were afraid and stressed due to the stringent lockdown and its restrictions on routine life as well as seeking basic or emergency medical services.

In a web-based survey conducted in China by Huang et al. [20], the prevalence of generalized anxiety disorders, depressive symptoms, and altered sleep quality was 35%, 20%, and 18%, respectively. Younger people and healthcare workers were the most vulnerable population. The study recommended limiting the time of receiving COVID-19-related information to less than 2 hours a day, focusing only on the necessary information, deferring from receiving too many harmful rumors, avoiding paying too much attention to outbreak information before going to sleep, exercising regularly, and maintain a regular rhythm of work and rest to ensure stress-free quality sleep [20]. During the swine flu pandemic in 2009-2010, various surveys studied the psychological status of victims and the general population for planning or modifying existing strategies for improving preventive measures to contain the outbreak [10,21]. Similarly, several scales were developed to assess COVID-19-related fear or peritraumatic stress, such as the CPDI scale in China, a 7-point Fear of COVID-19 scale in Turkey, and Bangla Fear of COVID-19 scale [9].

In an online survey by Qiu et al. [2], using the CPDI scale in February 2020, 52,730 respondents were from China, Hong Kong, Macau, and Taiwan, and the mean CPDI score of the sample population was 23.65. Almost 35% of the respondents had experienced psychological distress during the pandemic (30% of the respondents' scores were between 28 and 51, suggesting their stress level to be mild-to-moderate, and 5% had scores ≥52, which correlated with severe distress). The study revealed that psychological distress level was also influenced by the availability of local medical resources, the efficiency of the regional public health system, and the prevention and control measures taken by the government against the epidemic situation. Contrary to high migrant workers in China near Shanghai, who were supposedly asymptomatic carriers of coronavirus, the CPDI score was not that high because of the best healthcare facility there. (2) In the current study, we used the CPDI scale and its standardized questionnaire as a validated tool to measure psychological distress. Upon categorizing the study participants according to the three levels of distress, it was observed that 36.3% reported feeling no to least distress (CPDI <28), approximately half perceived a mild-to-moderate level of distress, and only 15.7% experienced severe distress (CPDI >51) while seeking care for their patients in the hospital during the COVID-19 pandemic.

Another large-scale online survey done in China (1,210 participants from 194 cities) in early 2020 assessed the psychological and mental health status by the Depression, Anxiety, and Stress Scale (DASS-21) [22]. That revealed a higher prevalence (53.8%) of moderate to severe psychological stress, and it was associated with female gender, student status, specific physical symptoms (e.g., myalgia, dizziness, coryza), and poor self-rated health status as the most vulnerable population. Among respondents, >70% were worried about their family members contracting COVID-19. The internet was the primary health information channel (93.5%), and 70% were satisfied with the amount of health information available through various sources. The measures suggested by the Chinese study to prevent psychological stress were to identify high-risk groups for early psychological interventions even via telemedicine services, smartphone-based psychoeducation through counseling, lectures, or other teaching activities, online portals or web-based applications, and information in a diagrammatic or audio format in simple language for illiterate individuals. The hospitals should provide resources for psychological support and accurate, crisp, authentic health information to patients and relatives to reduce the impact of rumors. Similarly, additional information on medicines or vaccines, routes of transmission, and updates on the actual data of infected cases and location (e.g., real-time, online tracking map) were associated with lower anxiety levels. This study also supported the need for relaxation exercises to counteract anxiety, and good infrastructures to produce adequate masks, soaps, alcohol-based hand rubs, and other personal protective equipment during the COVID-19-like pandemic.

The logistic regression in our study showed significantly higher odds of mild-to-moderate to severe stress in the younger age group of <45 years and the upper class of SES strata. However, gender, education, and residence did not exhibit significant differences in stress levels when compared comparatively.

The study had a minor limitation of depicting a single-center observation. But in the era of online surveys, we need more such offline and direct in-depth interviews regarding the major concerns and psychosocial aspects related to COVID-19 pandemic covering more diverse populations from distinct representative parts of the country. Similar problem-oriented studies might be helpful to policymakers and healthcare systems to develop adequate emergency preparedness and proper mitigation measures against such odds in the future.

## Conclusions

The COVID-19 pandemic was one of the worst global nightmares, and its impact was more obvious in developing nations, hitting hard not just their national healthcare systems, but severely compromising other emergency and routine medical services. This study reflected the real-time experience, awareness, concerns, and perception of various levels of stress from the viewpoint of caregivers of the patients attending to emergency facilities during the pandemic and lockdown constraints. The majority of them were afraid to go out of their houses (even to seek any hospital’s services) during lockdown restrictions, and access was difficult due to various reasons, such as the lack of transport facilities, poor socioeconomic support for non-COVID-19 illnesses, and healthcare system prioritization towards COVID-19 control. Almost half of the participant caregivers perceived mild-to-moderate distress and 16% experienced severe psychological distress while seeking treatment during the crunch. There was a significant association of stress level with age group, education, and socioeconomic class. Logistic regression revealed that in comparison to no stress, the odds of having moderate to severe stress were significantly higher among the younger-age patients <45 years (OR=1.515, p=0.024), and the upper SES class had higher odds (OR=1.613, p<0.001) of having moderate-to-severe stress than the lower class.
